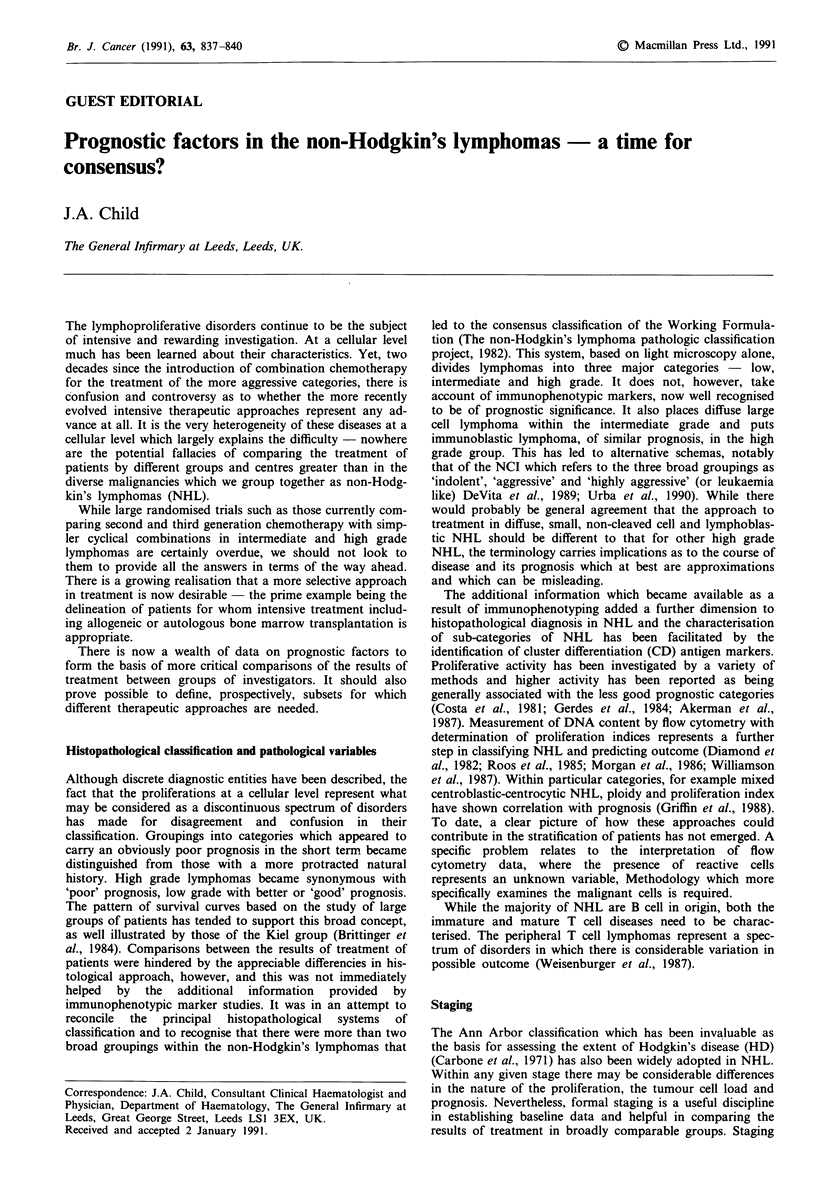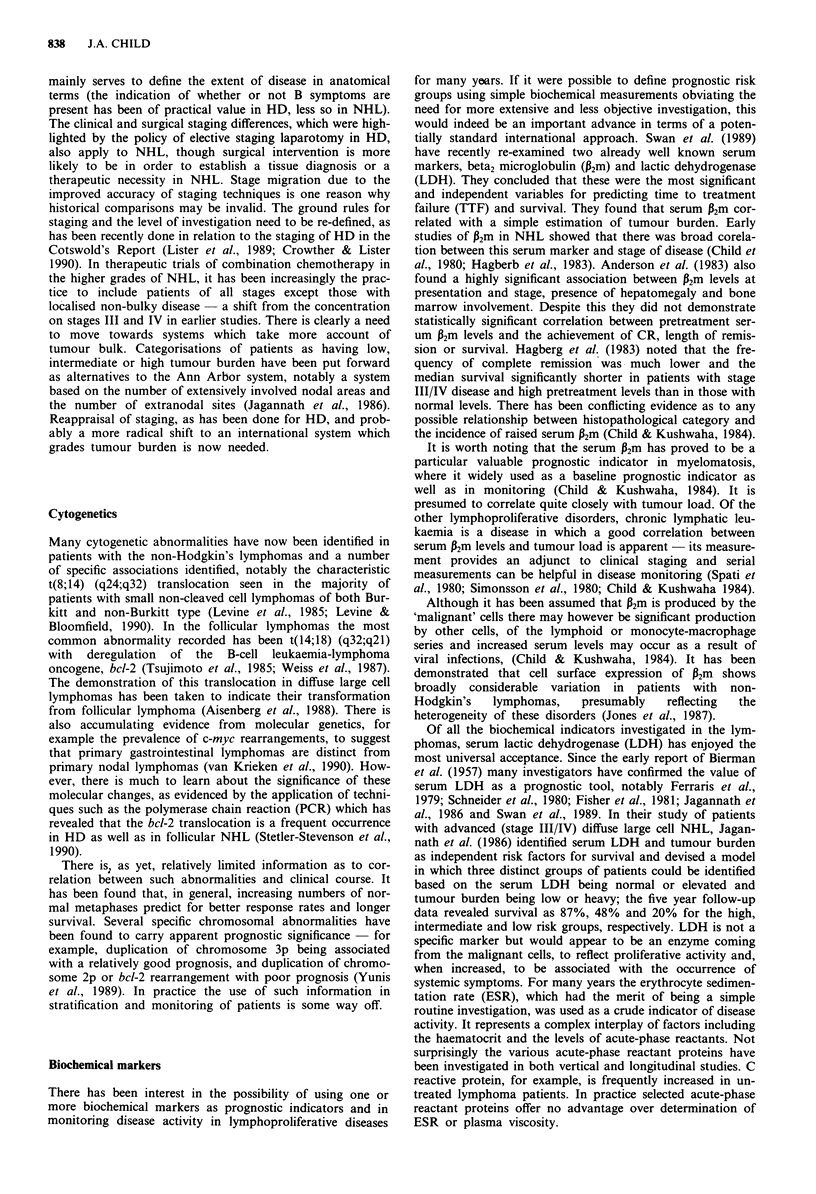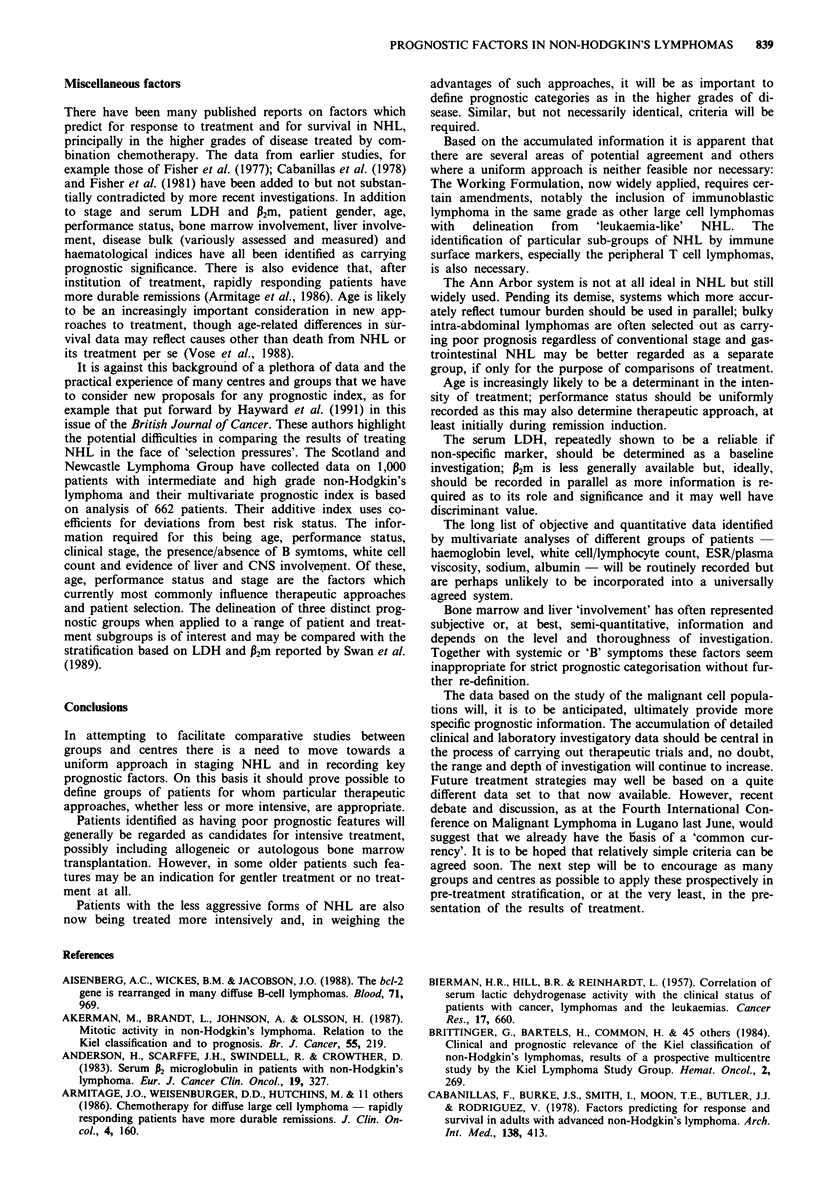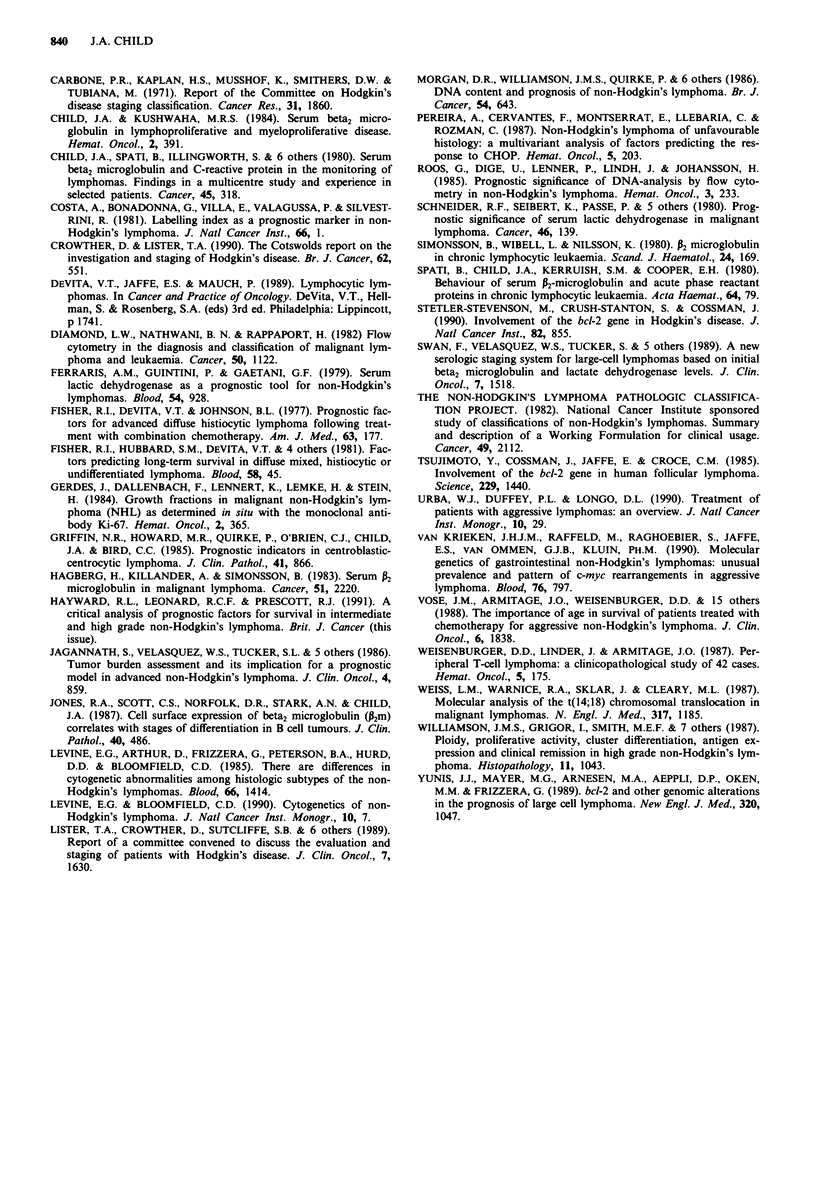# Prognostic factors in the non-Hodgkin's lymphomas--a time for consensus?

**DOI:** 10.1038/bjc.1991.186

**Published:** 1991-06

**Authors:** J. A. Child


Br. J. Cancer (1991), 63, 837–840 © Macmillan Press Ltd., 1991

GUEST EDITORIAL

### Prognostic factors in the non-Hodgkin's lymphomas — a time for consensus?

J.A. Child

*The General Infirmary at Leeds, Leeds, UK.*

The lymphoproliferative disorders continue to be the subject of intensive and rewarding investigation. At a cellular level much has been learned about their characteristics. Yet, two decades since the introduction of combination chemotherapy for the treatment of the more aggressive categories, there is confusion and controversy as to whether the more recently evolved intensive therapeutic approaches represent any advance at all. It is the very heterogeneity of these diseases at a cellular level which largely explains the difficulty — nowhere are the potential fallacies of comparing the treatment of patients by different groups and centres greater than in the diverse malignancies which we group together as non-Hodgkin's lymphomas (NHL).

While large randomised trials such as those currently comparing second and third generation chemotherapy with simpler cyclical combinations in intermediate and high grade lymphomas are certainly overdue, we should not look to them to provide all the answers in terms of the way ahead. There is a growing realisation that a more selective approach in treatment is now desirable — the prime example being the delineation of patients for whom intensive treatment including allogeneic or autologous bone marrow transplantation is appropriate.

There is now a wealth of data on prognostic factors to form the basis of more critical comparisons of the results of treatment between groups of investigators. It should also prove possible to define, prospectively, subsets for which different therapeutic approaches are needed.

### Histopathological classification and pathological variables

Although discrete diagnostic entities have been described, the fact that the proliferations at a cellular level represent what may be considered as a discontinuous spectrum of disorders has made for disagreement and confusion in their classification. Groupings into categories which appeared to carry an obviously poor prognosis in the short term became distinguished from those with a more protracted natural history. High grade lymphomas became synonymous with 'poor' prognosis, low grade with better or 'good' prognosis. The pattern of survival curves based on the study of large groups of patients has tended to support this broad concept, as well illustrated by those of the Kiel group (Brittinger *et al.*, 1984). Comparisons between the results of treatment of patients were hindered by the appreciable differencies in histological approach, however, and this was not immediately helped by the additional information provided by immunophenotypic marker studies. It was in an attempt to reconcile the principal histopathological systems of classification and to recognise that there were more than two broad groupings within the non-Hodgkin's lymphomas that led to the consensus classification of the Working Formulation (The non-Hodgkin's lymphoma pathologic classification project, 1982). This system, based on light microscopy alone, divides lymphomas into three major categories — low, intermediate and high grade. It does not, however, take account of immunophenotypic markers, now well recognised to be of prognostic significance. It also places diffuse large cell lymphoma within the intermediate grade and puts immunoblastic lymphoma, of similar prognosis, in the high grade group. This has led to alternative schemas, notably that of the NCI which refers to the three broad groupings as 'indolent', 'aggressive' and 'highly aggressive' (or leukaemia like) DeVita *et al.*, 1989; Urba *et al.*, 1990). While there would probably be general agreement that the approach to treatment in diffuse, small, non-cleaved cell and lymphoblastic NHL should be different to that for other high grade NHL, the terminology carries implications as to the course of disease and its prognosis which at best are approximations and which can be misleading.

The additional information which became available as a result of immunophenotyping added a further dimension to histopathological diagnosis in NHL and the characterisation of sub-categories of NHL has been facilitated by the identification of cluster differentiation (CD) antigen markers. Proliferative activity has been investigated by a variety of methods and higher activity has been reported as being generally associated with the less good prognostic categories (Costa *et al.*, 1981; Gerdes *et al.*, 1984; Akerman *et al.*, 1987). Measurement of DNA content by flow cytometry with determination of proliferation indices represents a further step in classifying NHL and predicting outcome (Diamond *et al.*, 1982; Roos *et al.*, 1985; Morgan *et al.*, 1986; Williamson *et al.*, 1987). Within particular categories, for example mixed centroblastic-centrocytic NHL, ploidy and proliferation index have shown correlation with prognosis (Griffin *et al.*, 1988). To date, a clear picture of how these approaches could contribute in the stratification of patients has not emerged. A specific problem relates to the interpretation of flow cytometry data, where the presence of reactive cells represents an unknown variable, Methodology which more specifically examines the malignant cells is required.

While the majority of NHL are B cell in origin, both the immature and mature T cell diseases need to be characterised. The peripheral T cell lymphomas represent a spectrum of disorders in which there is considerable variation in possible outcome (Weisenburger *et al.*, 1987).

### Staging

The Ann Arbor classification which has been invaluable as the basis for assessing the extent of Hodgkin's disease (HD) (Carbone *et al.*, 1971) has also been widely adopted in NHL. Within any given stage there may be considerable differences in the nature of the proliferation, the tumour cell load and prognosis. Nevertheless, formal staging is a useful discipline in establishing baseline data and helpful in comparing the results of treatment in broadly comparable groups. Staging

Correspondence: J.A. Child, Consultant Clinical Haematologist and Physician, Department of Haematology, The General Infirmary at Leeds, Great George Street, Leeds LS1 3EX, UK.
Received and accepted 2 January 1991.

mainly serves to define the extent of disease in anatomical terms (the indication of whether or not B symptoms are present has been of practical value in HD, less so in NHL). The clinical and surgical staging differences, which were highlighted by the policy of elective staging laparotomy in HD, also apply to NHL, though surgical intervention is more likely to be in order to establish a tissue diagnosis or a therapeutic necessity in NHL. Stage migration due to the improved accuracy of staging techniques is one reason why historical comparisons may be invalid. The ground rules for staging and the level of investigation need to be re-defined, as has been recently done in relation to the staging of HD in the Cotswold's Report (Lister *et al.*, 1989; Crowther & Lister 1990). In therapeutic trials of combination chemotherapy in the higher grades of NHL, it has been increasingly the practice to include patients of all stages except those with localised non-bulky disease — a shift from the concentration on stages III and IV in earlier studies. There is clearly a need to move towards systems which take more account of tumour bulk. Categorisations of patients as having low, intermediate or high tumour burden have been put forward as alternatives to the Ann Arbor system, notably a system based on the number of extensively involved nodal areas and the number of extranodal sites (Jagannath *et al.*, 1986). Reappraisal of staging, as has been done for HD, and probably a more radical shift to an international system which grades tumour burden is now needed.

### Cytogenetics

Many cytogenetic abnormalities have now been identified in patients with the non-Hodgkin's lymphomas and a number of specific associations identified, notably the characteristic t(8;14) (q24;q32) translocation seen in the majority of patients with small non-cleaved cell lymphomas of both Burkitt and non-Burkitt type (Levine *et al.*, 1985; Levine & Bloomfield, 1990). In the follicular lymphomas the most common abnormality recorded has been t(14;18) (q32;q21) with deregulation of the B-cell leukaemia-lymphoma oncogene, *bcl*-2 (Tsujimoto *et al.*, 1985; Weiss *et al.*, 1987). The demonstration of this translocation in diffuse large cell lymphomas has been taken to indicate their transformation from follicular lymphoma (Aisenberg *et al.*, 1988). There is also accumulating evidence from molecular genetics, for example the prevalence of c-*myc* rearrangements, to suggest that primary gastrointestinal lymphomas are distinct from primary nodal lymphomas (van Krieken *et al.*, 1990). However, there is much to learn about the significance of these molecular changes, as evidenced by the application of techniques such as the polymerase chain reaction (PCR) which has revealed that the *bcl*-2 translocation is a frequent occurrence in HD as well as in follicular NHL (Stetler-Stevenson *et al.*, 1990).

There is, as yet, relatively limited information as to correlation between such abnormalities and clinical course. It has been found that, in general, increasing numbers of normal metaphases predict for better response rates and longer survival. Several specific chromosomal abnormalities have been found to carry apparent prognostic significance — for example, duplication of chromosome 3p being associated with a relatively good prognosis, and duplication of chromosome 2p or *bcl*-2 rearrangement with poor prognosis (Yunis *et al.*, 1989). In practice the use of such information in stratification and monitoring of patients is some way off.

### Biochemical markers

There has been interest in the possibility of using one or more biochemical markers as prognostic indicators and in monitoring disease activity in lymphoproliferative diseases for many years. If it were possible to define prognostic risk groups using simple biochemical measurements obviating the need for more extensive and less objective investigation, this would indeed be an important advance in terms of a potentially standard international approach. Swan *et al.* (1989) have recently re-examined two already well known serum markers, beta$_2$ microglobulin ($\beta_2$m) and lactic dehydrogenase (LDH). They concluded that these were the most significant and independent variables for predicting time to treatment failure (TTF) and survival. They found that serum $\beta_2$m correlated with a simple estimation of tumour burden. Early studies of $\beta_2$m in NHL showed that there was broad corelation between this serum marker and stage of disease (Child *et al.*, 1980; Hagberb *et al.*, 1983). Anderson *et al.* (1983) also found a highly significant association between $\beta_2$m levels at presentation and stage, presence of hepatomegaly and bone marrow involvement. Despite this they did not demonstrate statistically significant correlation between pretreatment serum $\beta_2$m levels and the achievement of CR, length of remission or survival. Hagberg *et al.* (1983) noted that the frequency of complete remission was much lower and the median survival significantly shorter in patients with stage III/IV disease and high pretreatment levels than in those with normal levels. There has been conflicting evidence as to any possible relationship between histopathological category and the incidence of raised serum $\beta_2$m (Child & Kushwaha, 1984).

It is worth noting that the serum $\beta_2$m has proved to be a particular valuable prognostic indicator in myelomatosis, where it widely used as a baseline prognostic indicator as well as in monitoring (Child & Kushwaha, 1984). It is presumed to correlate quite closely with tumour load. Of the other lymphoproliferative disorders, chronic lymphatic leukaemia is a disease in which a good correlation between serum $\beta_2$m levels and tumour load is apparent — its measurement provides an adjunct to clinical staging and serial measurements can be helpful in disease monitoring (Spati *et al.*, 1980; Simonsson *et al.*, 1980; Child & Kushwaha 1984).

Although it has been assumed that $\beta_2$m is produced by the 'malignant' cells there may however be significant production by other cells, of the lymphoid or monocyte-macrophage series and increased serum levels may occur as a result of viral infections, (Child & Kushwaha, 1984). It has been demonstrated that cell surface expression of $\beta_2$m shows broadly considerable variation in patients with non-Hodgkin's lymphomas, presumably reflecting the heterogeneity of these disorders (Jones *et al.*, 1987).

Of all the biochemical indicators investigated in the lymphomas, serum lactic dehydrogenase (LDH) has enjoyed the most universal acceptance. Since the early report of Bierman *et al.* (1957) many investigators have confirmed the value of serum LDH as a prognostic tool, notably Ferraris *et al.*, 1979; Schneider *et al.*, 1980; Fisher *et al.*, 1981; Jagannath *et al.*, 1986 and Swan *et al.*, 1989. In their study of patients with advanced (stage III/IV) diffuse large cell NHL, Jagannath *et al.* (1986) identified serum LDH and tumour burden as independent risk factors for survival and devised a model in which three distinct groups of patients could be identified based on the serum LDH being normal or elevated and tumour burden being low or heavy; the five year follow-up data revealed survival as 87%, 48% and 20% for the high, intermediate and low risk groups, respectively. LDH is not a specific marker but would appear to be an enzyme coming from the malignant cells, to reflect proliferative activity and, when increased, to be associated with the occurrence of systemic symptoms. For many years the erythrocyte sedimentation rate (ESR), which had the merit of being a simple routine investigation, was used as a crude indicator of disease activity. It represents a complex interplay of factors including the haematocrit and the levels of acute-phase reactants. Not surprisingly the various acute-phase reactant proteins have been investigated in both vertical and longitudinal studies. C reactive protein, for example, is frequently increased in untreated lymphoma patients. In practice selected acute-phase reactant proteins offer no advantage over determination of ESR or plasma viscosity.

**Miscellaneous factors**

There have been many published reports on factors which predict for response to treatment and for survival in NHL, principally in the higher grades of disease treated by combination chemotherapy. The data from earlier studies, for example those of Fisher *et al.* (1977); Cabanillas *et al.* (1978) and Fisher *et al.* (1981) have been added to but not substantially contradicted by more recent investigations. In addition to stage and serum LDH and $\beta_2$m, patient gender, age, performance status, bone marrow involvement, liver involvement, disease bulk (variously assessed and measured) and haematological indices have all been identified as carrying prognostic significance. There is also evidence that, after institution of treatment, rapidly responding patients have more durable remissions (Armitage *et al.*, 1986). Age is likely to be an increasingly important consideration in new approaches to treatment, though age-related differences in survival data may reflect causes other than death from NHL or its treatment per se (Vose *et al.*, 1988).

It is against this background of a plethora of data and the practical experience of many centres and groups that we have to consider new proposals for any prognostic index, as for example that put forward by Hayward *et al.* (1991) in this issue of the *British Journal of Cancer*. These authors highlight the potential difficulties in comparing the results of treating NHL in the face of 'selection pressures'. The Scotland and Newcastle Lymphoma Group have collected data on 1,000 patients with intermediate and high grade non-Hodgkin's lymphoma and their multivariate prognostic index is based on analysis of 662 patients. Their additive index uses coefficients for deviations from best risk status. The information required for this being age, performance status, clinical stage, the presence/absence of B symtoms, white cell count and evidence of liver and CNS involvement. Of these, age, performance status and stage are the factors which currently most commonly influence therapeutic approaches and patient selection. The delineation of three distinct prognostic groups when applied to a range of patient and treatment subgroups is of interest and may be compared with the stratification based on LDH and $\beta_2$m reported by Swan *et al.* (1989).

**Conclusions**

In attempting to facilitate comparative studies between groups and centres there is a need to move towards a uniform approach in staging NHL and in recording key prognostic factors. On this basis it should prove possible to define groups of patients for whom particular therapeutic approaches, whether less or more intensive, are appropriate.

Patients identified as having poor prognostic features will generally be regarded as candidates for intensive treatment, possibly including allogeneic or autologous bone marrow transplantation. However, in some older patients such features may be an indication for gentler treatment or no treatment at all.

Patients with the less aggressive forms of NHL are also now being treated more intensively and, in weighing the advantages of such approaches, it will be as important to define prognostic categories as in the higher grades of disease. Similar, but not necessarily identical, criteria will be required.

Based on the accumulated information it is apparent that there are several areas of potential agreement and others where a uniform approach is neither feasible nor necessary: The Working Formulation, now widely applied, requires certain amendments, notably the inclusion of immunoblastic lymphoma in the same grade as other large cell lymphomas with delineation from 'leukaemia-like' NHL. The identification of particular sub-groups of NHL by immune surface markers, especially the peripheral T cell lymphomas, is also necessary.

The Ann Arbor system is not at all ideal in NHL but still widely used. Pending its demise, systems which more accurately reflect tumour burden should be used in parallel; bulky intra-abdominal lymphomas are often selected out as carrying poor prognosis regardless of conventional stage and gastrointestinal NHL may be better regarded as a separate group, if only for the purpose of comparisons of treatment.

Age is increasingly likely to be a determinant in the intensity of treatment; performance status should be uniformly recorded as this may also determine therapeutic approach, at least initially during remission induction.

The serum LDH, repeatedly shown to be a reliable if non-specific marker, should be determined as a baseline investigation; $\beta_2$m is less generally available but, ideally, should be recorded in parallel as more information is required as to its role and significance and it may well have discriminant value.

The long list of objective and quantitative data identified by multivariate analyses of different groups of patients — haemoglobin level, white cell/lymphocyte count, ESR/plasma viscosity, sodium, albumin — will be routinely recorded but are perhaps unlikely to be incorporated into a universally agreed system.

Bone marrow and liver 'involvement' has often represented subjective or, at best, semi-quantitative, information and depends on the level and thoroughness of investigation. Together with systemic or 'B' symptoms these factors seem inappropriate for strict prognostic categorisation without further re-definition.

The data based on the study of the malignant cell populations will, it is to be anticipated, ultimately provide more specific prognostic information. The accumulation of detailed clinical and laboratory investigatory data should be central in the process of carrying out therapeutic trials and, no doubt, the range and depth of investigation will continue to increase. Future treatment strategies may well be based on a quite different data set to that now available. However, recent debate and discussion, as at the Fourth International Conference on Malignant Lymphoma in Lugano last June, would suggest that we already have the basis of a 'common currency'. It is to be hoped that relatively simple criteria can be agreed soon. The next step will be to encourage as many groups and centres as possible to apply these prospectively in pre-treatment stratification, or at the very least, in the presentation of the results of treatment.

**References**

AISENBERG, A.C., WICKES, B.M. & JACOBSON, J.O. (1988). The *bcl*-2 gene is rearranged in many diffuse B-cell lymphomas. *Blood*, **71**, 969.

AKERMAN, M., BRANDT, L., JOHNSON, A. & OLSSON, H. (1987). Mitotic activity in non-Hodgkin's lymphoma. Relation to the Kiel classification and to prognosis. *Br. J. Cancer*, **55**, 219.

ANDERSON, H., SCARFFE, J.H., SWINDELL, R. & CROWTHER, D. (1983). Serum $\beta_2$ microglobulin in patients with non-Hodgkin's lymphoma. *Eur. J. Cancer Clin. Oncol.*, **19**, 327.

ARMITAGE, J.O., WEISENBURGER, D.D., HUTCHINS, M. & 11 others (1986). Chemotherapy for diffuse large cell lymphoma — rapidly responding patients have more durable remissions. *J. Clin. Oncol.*, **4**, 160.

BIERMAN, H.R., HILL, B.R. & REINHARDT, L. (1957). Correlation of serum lactic dehydrogenase activity with the clinical status of patients with cancer, lymphomas and the leukaemias. *Cancer Res.*, **17**, 660.

BRITTINGER, G., BARTELS, H., COMMON, H. & 45 others (1984). Clinical and prognostic relevance of the Kiel classification of non-Hodgkin's lymphomas, results of a prospective multicentre study by the Kiel Lymphoma Study Group. *Hemat. Oncol.*, **2**, 269.

CABANILLAS, F., BURKE, J.S., SMITH, I., MOON, T.E., BUTLER, J.J. & RODRIGUEZ, V. (1978). Factors predicting for response and survival in adults with advanced non-Hodgkin's lymphoma. *Arch. Int. Med.*, **138**, 413.

CARBONE, P.R., KAPLAN, H.S., MUSSHOF, K., SMITHERS, D.W. & TUBIANA, M. (1971). Report of the Committee on Hodgkin's disease staging classification. *Cancer Res.*, **31**, 1860.
CHILD, J.A. & KUSHWAHA, M.R.S. (1984). Serum beta$_2$ microglobulin in lymphoproliferative and myeloproliferative disease. *Hemat. Oncol.*, **2**, 391.
CHILD, J.A., SPATI, B., ILLINGWORTH, S. & 6 others (1980). Serum beta$_2$ microglobulin and C-reactive protein in the monitoring of lymphomas. Findings in a multicentre study and experience in selected patients. *Cancer*, **45**, 318.
COSTA, A., BONADONNA, G., VILLA, E., VALAGUSSA, P. & SILVESTRINI, R. (1981). Labelling index as a prognostic marker in non-Hodgkin's lymphoma. *J. Natl Cancer Inst.*, **66**, 1.
CROWTHER, D. & LISTER, T.A. (1990). The Cotswolds report on the investigation and staging of Hodgkin's disease. *Br. J. Cancer*, **62**, 551.
DEVITA, V.T., JAFFE, E.S. & MAUCH, P. (1989). Lymphocytic lymphomas. In *Cancer and Practice of Oncology*. DeVita, V.T., Hellman, S. & Rosenberg, S.A. (eds) 3rd ed. Philadelphia: Lippincott, p 1741.
DIAMOND, L.W., NATHWANI, B. N. & RAPPAPORT, H. (1982) Flow cytometry in the diagnosis and classification of malignant lymphoma and leukaemia. *Cancer*, **50**, 1122.
FERRARIS, A.M., GUINTINI, P. & GAETANI, G.F. (1979). Serum lactic dehydrogenase as a prognostic tool for non-Hodgkin's lymphomas. *Blood*, **54**, 928.
FISHER, R.I., DEVITA, V.T. & JOHNSON, B.L. (1977). Prognostic factors for advanced diffuse histiocytic lymphoma following treatment with combination chemotherapy. *Am. J. Med.*, **63**, 177.
FISHER, R.I., HUBBARD, S.M., DEVITA, V.T. & 4 others (1981). Factors predicting long-term survival in diffuse mixed, histiocytic or undifferentiated lymphoma. *Blood*, **58**, 45.
GERDES, J., DALLENBACH, F., LENNERT, K., LEMKE, H. & STEIN, H. (1984). Growth fractions in malignant non-Hodgkin's lymphoma (NHL) as determined *in situ* with the monoclonal antibody Ki-67. *Hemat. Oncol.*, **2**, 365.
GRIFFIN, N.R., HOWARD, M.R., QUIRKE, P., O'BRIEN, C.J., CHILD, J.A. & BIRD, C.C. (1985). Prognostic indicators in centroblastic-centrocytic lymphoma. *J. Clin. Pathol.*, **41**, 866.
HAGBERG, H., KILLANDER, A. & SIMONSSON, B. (1983). Serum $\beta_2$ microglobulin in malignant lymphoma. *Cancer*, **51**, 2220.
HAYWARD, R.L., LEONARD, R.C.F. & PRESCOTT, R.J. (1991). A critical analysis of prognostic factors for survival in intermediate and high grade non-Hodgkin's lymphoma. *Brit. J. Cancer* (this issue).
JAGANNATH, S., VELASQUEZ, W.S., TUCKER, S.L. & 5 others (1986). Tumor burden assessment and its implication for a prognostic model in advanced non-Hodgkin's lymphoma. *J. Clin. Oncol.*, **4**, 859.
JONES, R.A., SCOTT, C.S., NORFOLK, D.R., STARK, A.N. & CHILD, J.A. (1987). Cell surface expression of beta$_2$ microglobulin ($\beta_2$m) correlates with stages of differentiation in B cell tumours. *J. Clin. Pathol.*, **40**, 486.
LEVINE, E.G., ARTHUR, D., FRIZZERA, G., PETERSON, B.A., HURD, D.D. & BLOOMFIELD, C.D. (1985). There are differences in cytogenetic abnormalities among histologic subtypes of the non-Hodgkin's lymphomas. *Blood*, **66**, 1414.
LEVINE, E.G. & BLOOMFIELD, C.D. (1990). Cytogenetics of non-Hodgkin's lymphoma. *J. Natl Cancer Inst. Monogr.*, **10**, 7.
LISTER, T.A., CROWTHER, D., SUTCLIFFE, S.B. & 6 others (1989). Report of a committee convened to discuss the evaluation and staging of patients with Hodgkin's disease. *J. Clin. Oncol.*, **7**, 1630.
MORGAN, D.R., WILLIAMSON, J.M.S., QUIRKE, P. & 6 others (1986). DNA content and prognosis of non-Hodgkin's lymphoma. *Br. J. Cancer*, **54**, 643.
PEREIRA, A., CERVANTES, F., MONTSERRAT, E., LLEBARIA, C. & ROZMAN, C. (1987). Non-Hodgkin's lymphoma of unfavourable histology: a multivariant analysis of factors predicting the response to CHOP. *Hemat. Oncol.*, **5**, 203.
ROOS, G., DIGE, U., LENNER, P., LINDH, J. & JOHANSSON, H. (1985). Prognostic significance of DNA-analysis by flow cytometry in non-Hodgkin's lymphoma. *Hemat. Oncol.*, **3**, 233.
SCHNEIDER, R.F., SEIBERT, K., PASSE, P. & 5 others (1980). Prognostic significance of serum lactic dehydrogenase in malignant lymphoma. *Cancer*, **46**, 139.
SIMONSSON, B., WIBELL, L. & NILSSON, K. (1980). $\beta_2$ microglobulin in chronic lymphocytic leukaemia. *Scand. J. Haematol.*, **24**, 169.
SPATI, B., CHILD, J.A., KERRUISH, S.M. & COOPER, E.H. (1980). Behaviour of serum $\beta_2$-microglobulin and acute phase reactant proteins in chronic lymphocytic leukaemia. *Acta Haemat.*, **64**, 79.
STETLER-STEVENSON, M., CRUSH-STANTON, S. & COSSMAN, J. (1990). Involvement of the *bcl*-2 gene in Hodgkin's disease. *J. Natl Cancer Inst.*, **82**, 855.
SWAN, F., VELASQUEZ, W.S., TUCKER, S. & 5 others (1989). A new serologic staging system for large-cell lymphomas based on initial beta$_2$ microglobulin and lactate dehydrogenase levels. *J. Clin. Oncol.*, **7**, 1518.
THE NON-HODGKIN'S LYMPHOMA PATHOLOGIC CLASSIFICATION PROJECT. (1982). National Cancer Institute sponsored study of classifications of non-Hodgkin's lymphomas. Summary and description of a Working Formulation for clinical usage. *Cancer*, **49**, 2112.
TSUJIMOTO, Y., COSSMAN, J., JAFFE, E. & CROCE, C.M. (1985). Involvement of the *bcl*-2 gene in human follicular lymphoma. *Science*, **229**, 1440.
URBA, W.J., DUFFEY, P.L. & LONGO, D.L. (1990). Treatment of patients with aggressive lymphomas: an overview. *J. Natl Cancer Inst. Monogr.*, **10**, 29.
VAN KRIEKEN, J.H.J.M., RAFFELD, M., RAGHOEBIER, S., JAFFE, E.S., VAN OMMEN, G.J.B., KLUIN, PH.M. (1990). Molecular genetics of gastrointestinal non-Hodgkin's lymphomas: unusual prevalence and pattern of *c-myc* rearrangements in aggressive lymphoma. *Blood*, **76**, 797.
VOSE, J.M., ARMITAGE, J.O., WEISENBURGER, D.D. & 15 others (1988). The importance of age in survival of patients treated with chemotherapy for aggressive non-Hodgkin's lymphoma. *J. Clin. Oncol.*, **6**, 1838.
WEISENBURGER, D.D., LINDER, J. & ARMITAGE, J.O. (1987). Peripheral T-cell lymphoma: a clinicopathological study of 42 cases. *Hemat. Oncol.*, **5**, 175.
WEISS, L.M., WARNICE, R.A., SKLAR, J. & CLEARY, M.L. (1987). Molecular analysis of the t(14;18) chromosomal translocation in malignant lymphomas. *N. Engl. J. Med.*, **317**, 1185.
WILLIAMSON, J.M.S., GRIGOR, I., SMITH, M.E.F. & 7 others (1987). Ploidy, proliferative activity, cluster differentiation, antigen expression and clinical remission in high grade non-Hodgkin's lymphoma. *Histopathology*, **11**, 1043.
YUNIS, J.J., MAYER, M.G., ARNESEN, M.A., AEPPLI, D.P., OKEN, M.M. & FRIZZERA, G. (1989). *bcl*-2 and other genomic alterations in the prognosis of large cell lymphoma. *New Engl. J. Med.*, **320**, 1047.